# Effect of forward moment on recovery motion against tripping

**DOI:** 10.1371/journal.pone.0298045

**Published:** 2024-02-14

**Authors:** Yasuhiro Akiyama, Aoto Nishizaki, Shogo Okamoto, Yoji Yamada

**Affiliations:** 1 Nagoya University, Chikusa-ku, Nagoya, Aichi, Japan; 2 Tokyo Metropolitan University, Hino, Tokyo, Japan; 3 National Institute of Technology, Toyota College, Toyota, Aichi, Japan; Polytechnic University of Marche: Universita Politecnica delle Marche, ITALY

## Abstract

Investigating the fall recovery motion mechanism is crucial to prevent fall injuries. Among the various parameters of motion and posture, the forward moment can be considered the representative parameter of the magnitude of tripping from a kinematic perspective. The effect of increasing the forward moment on the recovery motion after tripping was investigated in this study. A tripping experiment was performed on a treadmill, and the recovery motion was observed. The forward moment was artificially increased using several approaches, such as pulling the torso, increasing gait speed, and increasing body mass. Factor analysis was performed to establish the relationship between the recovery motion parameters and forward moment. The distribution of the factor scores implied the uniqueness of the recovery motion of the pull condition. Although the forward moment temporarily increased, it was compensated quickly. The other conditions and factors indicated qualitative similarity of the recovery motion among the different conditions. This study demonstrates that the recovery motion after tripping is robust against an increase in forward moment, regardless of the method used to increase the forward moment. The investigation of reaction motion pattern enables validation of the recovery motion and falling posture estimation. Such fall simulations will facilitate the development of a method of fall prevention and mitigation.

## Introduction

Prevention and mitigation of falls are important because falls frequently cause severe injuries and medical costs [1, 2]. Thus, the fall mechanism and parameters affecting recovery motion have been extensively investigated to extract fall risk factors [3–7]. However, the complex dependences among these parameters make it difficult to investigate the effects of each parameter respectively.

A basic method of investigating trips and falls involves inducing trips in a walking lane using obstacles [8, 9]. A common method involves positioning a flat plate, approximately 10 cm in height, above the walking lane to induce tripping in the walking subject [10, 11]. Another approach is to attach a cable to the ankle and pull it from behind [12]. Various recovery motions can be observed owing to the subject attributes, gait speed, and trip timing [13]. Simulated tripping has been performed on a treadmill to observe recovery motion [14–16]. Gait speed can be controlled on a treadmill more easily than in a walking lane. However, the method of applying gait perturbation sometimes differs from that used in overground walking lanes. For instance, applying a tripping-like perturbation can be achieved by pulling the leg from behind using a cable attached to the ankle, as described in Cordero et al. (2003) [17]. Thus, we developed a tripping treadmill that induces actual tripping using a horizontal bar moving along with the belt on the treadmill to observe realistic fall motion [18].

Previous studies have revealed the effects of several physical and gait parameters on the recovery motion. For example, some parameters, such as muscle weakness and reaction time, increase or decrease the risk of falls [5, 6]. Furthermore, a decrease in the ability of aged people to recover from tripping has been reported [7, 19, 20]. The effect of comfortable gait speed has been investigated as a specific parameter related to fall risk [6, 7, 19]. Some of these studies have revealed an increase in fall risk at a faster gait speed. However, other studies have indicated that the relationship between gait speed and stability is not simple because of the compensatory motion [21, 22]. Thus, a method of independently evaluating the effects of gait parameters has been proposed [23].

Based on kinematics, gait stability can be analyzed using the angular momentum [24, 25]. Thus, the fall recovery motion can be determined as the process controlling the forward moment generated by tripping. Pijnappels et al. analyzed the difference in recovery motion between fallers and non-fallers from the perspective of the forward moment [26]. The results indicated the importance of the ground reaction force of the stance foot during the swing phase of the recovery step to compensate for the forward moment. To some degree, an increase in forward moment forces the recovery motion to be more dynamic (i.e., increases in step length and body inclination increase the forward moment). Furthermore, an increase in the forward moment may change the structure of the recovery motion. We hypothesized that an increase in the forward moment would cause a qualitative difference in the recovery motion.

In this study, a treadmill experiment was performed to observe the recovery motion after tripping. The forward moment acting on the subject was artificially increased using various methods such as pulling the torso using a wire, increasing gait speed, and increasing the body mass using a vest with weights. The proposed method enabled the forward moment to be increased without changing the physical ability of the subject. This approach provides a novel means of investigating the effect of the forward moment independently and extracting the qualitative changes in the recovery motion against tripping.

## Methods

The experiment was conducted with approval from the Institutional Review Board of Nagoya University (approval number 20–6).

### Apparatus

**Fig 1:**
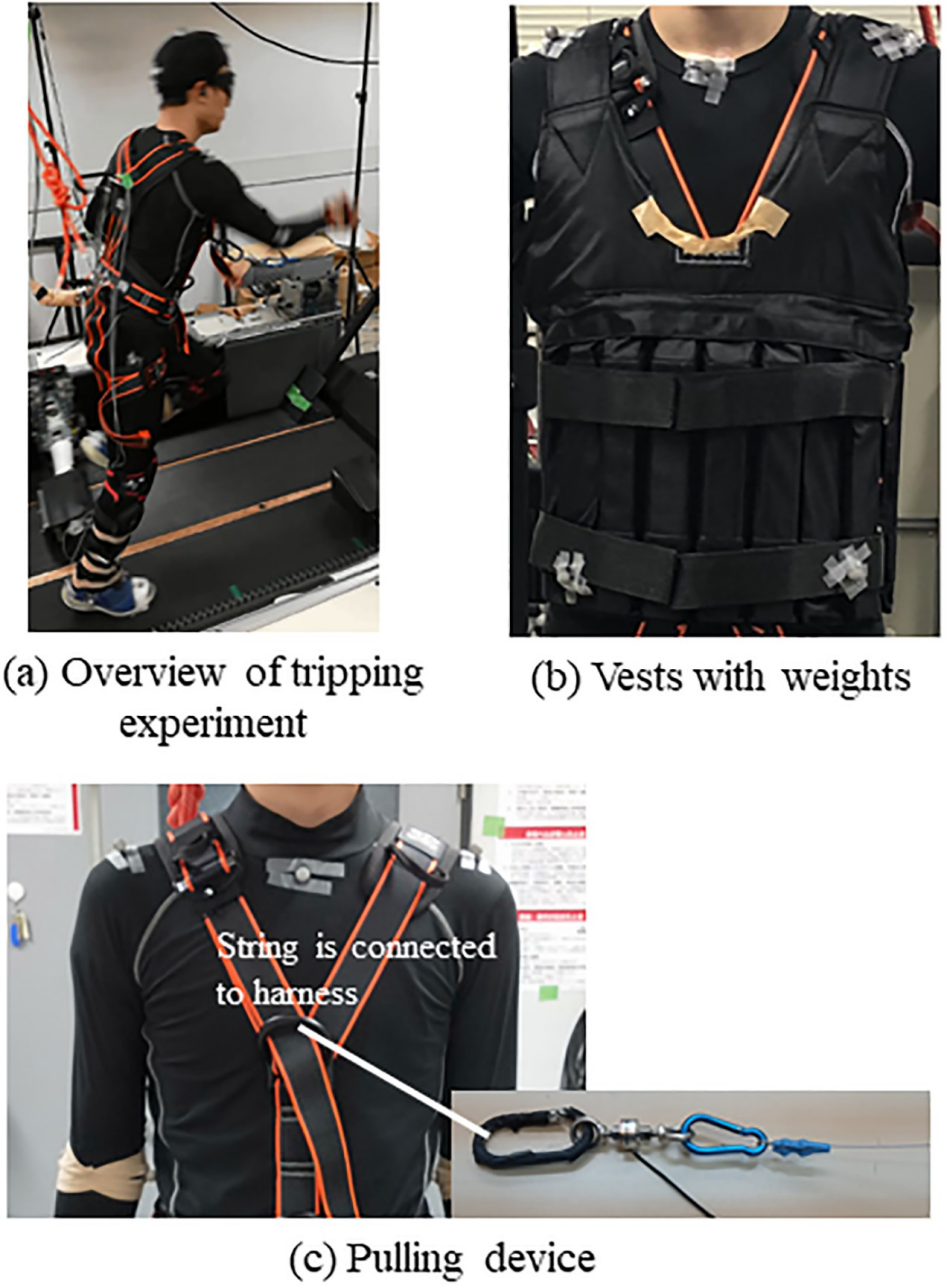
Experimental setups. (a) Overview of tripping experiment (b) Vests with weights (c) Pulling device.

The configuration of the tripping and observation system is illustrated in Fig 1. The experiment was performed on a double-belt treadmill (Ohtake Root Kogyo Co. Ltd., Japan). The perturbation mechanism consisted of two aluminum tripping bars and actuated linear sliders (e-Valley, Japan). The tripping bars were fixed sideways relative to the linear sliders located on the left and right sides and moved in a forward/backward direction at the same speed as the treadmill belt. The feet of the subjects hit metal plates fixed to the tripping bars. The vertical position of the top of metal plate was 15 cm in height. The height of the obstacle was within the range typically used in tripping experiments [4, 27]. An overview of the tripping experiment is provided in Fig 1(a). The motion of the subjects was recorded using a seven-camera motion capture system (OptiTrack, NaturalPoint Inc., US). The positions of the 22 markers attached to each subject were determined based on a set of critical markers [28].

The subjects wore vests, in which metal plates could be attached, to increase the forward rotation moment after tripping (Fig 1(b)). Furthermore, a device that pulls a string (e-Valley, Japan) was placed in front of the treadmill. The end of the string was fixed to the chest of the vest to increase the forward rotation moment by pulling the string simultaneously with the motion of the tripping bar. The pulling mechanism is presented in Fig 1(c).

The positions of the subjects were measured using a laser range finder (UST- 20LX, Hokuyo Automatic Co., Ltd., Japan) located in front of the belt. The gait phase of each subject was estimated by detecting the timing of heel contact (HC) that was measured using a four-foot switch (FSR UX 406, Interlink Electronics Inc., CA, USA) fixed under the heels and toes of the subject. To trip the subjects at the intended gait phase, the tripping bar was set to move at a specific time that was calculated from the estimated gait phase, position of the subject, and speed of the belt.

The subjects wore half-covered goggles and headphones with noise to mask the view and sound of the approaching tripping bar. Furthermore, subjects wore safety harnesses and plastic protectors. The harness was connected to the frame above the treadmill.

### Protocol

Eight healthy male adult university students without gait disorders or pain during walking were recruited. The mean and standard deviation of the age, height, and weight of the subjects were 23.7±0.5 years, 173.1±4.6 cm, and 64.9±5.2 kg, respectively.

Written consent was obtained from each subject after the explanation of the experimental procedure. The subjects wore well-fitting sportswear and shoes in which foot switches were installed. Reflective markers, vests, and safety gears were also attached. In the practice trials, the subjects walked on the treadmill for 5 min at 3.6 and 4.2 km/h, wearing half-covered goggles and headphones. Subsequently, several tripping trials were performed to practice and test the safety of the devices. Then, the recording session commenced.

The subjects tripped once in each trial, and their recovery motions after tripping were recorded. The side and timing of the tripping leg were generated arbitrarily to prevent anticipation. The subjects were instructed to recover from tripping and continue walking. The trip timing was controlled such that tripping occurred in the early to middle swing phase in all trials. Tripping during this gait phase is expected to result in the use of the elevating strategy [13, 29]. In the elevating strategy, the subjects moved the tripped leg forward to overcome the tripping bar; this step was called the first recovery step. The successive step was designated as the second recovery step. The first and second recovery steps were recorded and analyzed. The treadmill was stopped after recovery, completing a single trial.

For each subject, four experimental conditions were conducted: normal, pull, fast, and heavy. The treadmill speed was set to 3.6 km/h for the normal, pull, and heavy conditions. For the fast condition, the treadmill speed was set to 4.2 km/h. Metal plates, with a total weight of 10 kg, were inserted into the vest for the heavy condition. A steel string was fixed to the chest of the subjects for the pull condition. Pre-tension was applied to the string to prevent slack. The pulling force was approximately 90 N, and the duration was 105 ms. However, it was difficult to reproduce the impulse exactly owing to the variability of the recovery motion of the subjects. The integral of the increase in the forward moment around the stance foot was approximately 10 Nm⋅s under all sets of conditions. This value corresponds to approximately 15%-25% of the forward moment exerted under normal tripping conditions.

Six trials were conducted under each set of conditions. Thus, 24 trials were recorded for each subject. The order of the trials for each set of conditions was executed arbitrarily among the subjects, and the side of the tripped leg was selected arbitrarily in every trial.

### Data processing

Motion data were recorded at 120 Hz and processed using a 6 Hz Butterworth filter [30, 31]. The joint angles and posture of the subjects were calculated by fitting a human model to the positions of the markers using the least-squares method. The biomechanical analysis software SIMM (MulsculoGraphics Inc., Evanston, IL, USA) was used for this process [28].

To calculate the forward moment acting around the center of mass (CoM), a 17-link human body model was developed. It included the head, neck, abdomen, trunk, pelvis, upper arms, forearms, hands, thighs, shanks, and feet. The model was equipped with hip, knee, ankle, shoulder, elbow, and lumbar joints. The total number of degrees of freedom of the joints was 21. The size, mass, and inertia of each link were scaled based on the height and body mass of each subject [32, 33]. The moment acting on each body link was calculated using a kinematic formula. The whole-body moment was the summation of the moments of all body links. It was important to normalize the whole-body moment by the gait speed, body mass, and height to analyze the qualitative changes in the recovery motion among the sets of conditions.

The timing of HC and toe-off of each foot was determined manually based on the video and marker trajectory. The effects of contact-side differences were disregarded. Therefore, the recorded data of trials in which the right leg tripped mirrored the motion of the left leg.

To extract the characteristics of the elevating strategy, three gait events and two motion phases were defined as follows. The hit time (HT) was defined as the time at which tripping occurred. The first step (FS) was defined as the time at which the subject stepped over the tripping bar with the tripped leg. The subsequent step performed by the recovery leg was defined as the second step (SS). The time intervals between the HT and FS and the FS and SS were determined as the first and second recovery phases, respectively.

To evaluate the recovery motion, the following parameters were determined. *Tr*_*ang*_ is the trunk inclination angle at the FS and SS. *Hip*_*ang*_ is the maximum hip flexion angle during each phase. *CoMv* is the CoM speed at the FS and SS in the vertical direction. *St*_*length*_ is the step length, which was determined as the distance between the heel marker positions of the subsequent steps in the traveling direction. *St*_*time*_ is the step time, which was determined as the time duration of each step. *Max*_*mom*_ is the maximum value of the forward moment normalized by the gait speed, body mass, and height during the first recovery phase. *Mean*_*mom*_ is the mean value of the forward moment normalized by the gait speed, body mass, and height during the first recovery phase. *Ptime*_*mom*_ is the time until the forward moment reaches the maximum value from HT. *HC*_*mom*_ is the forward moment at the FS normalized by the gait speed, body mass, and height.

The first and third quartiles and median of each parameter were calculated for each set of conditions. The correlation coefficients among the recovery motion parameters were calculated to evaluate the relationships between the parameters. Factor analysis (FA) was performed to determine the factors that represented the recovery motion and to analyze the relationships among the recovery parameters. In particular, we focused on the relationships between the motion and moment parameters. The differences in the FA scores among the sets of conditions were compared using the Mann–Whitney *u*-test with Bonferroni’s method. The threshold for statistical significance was set to *p* < 0.05.

## Results

**Fig 2:**
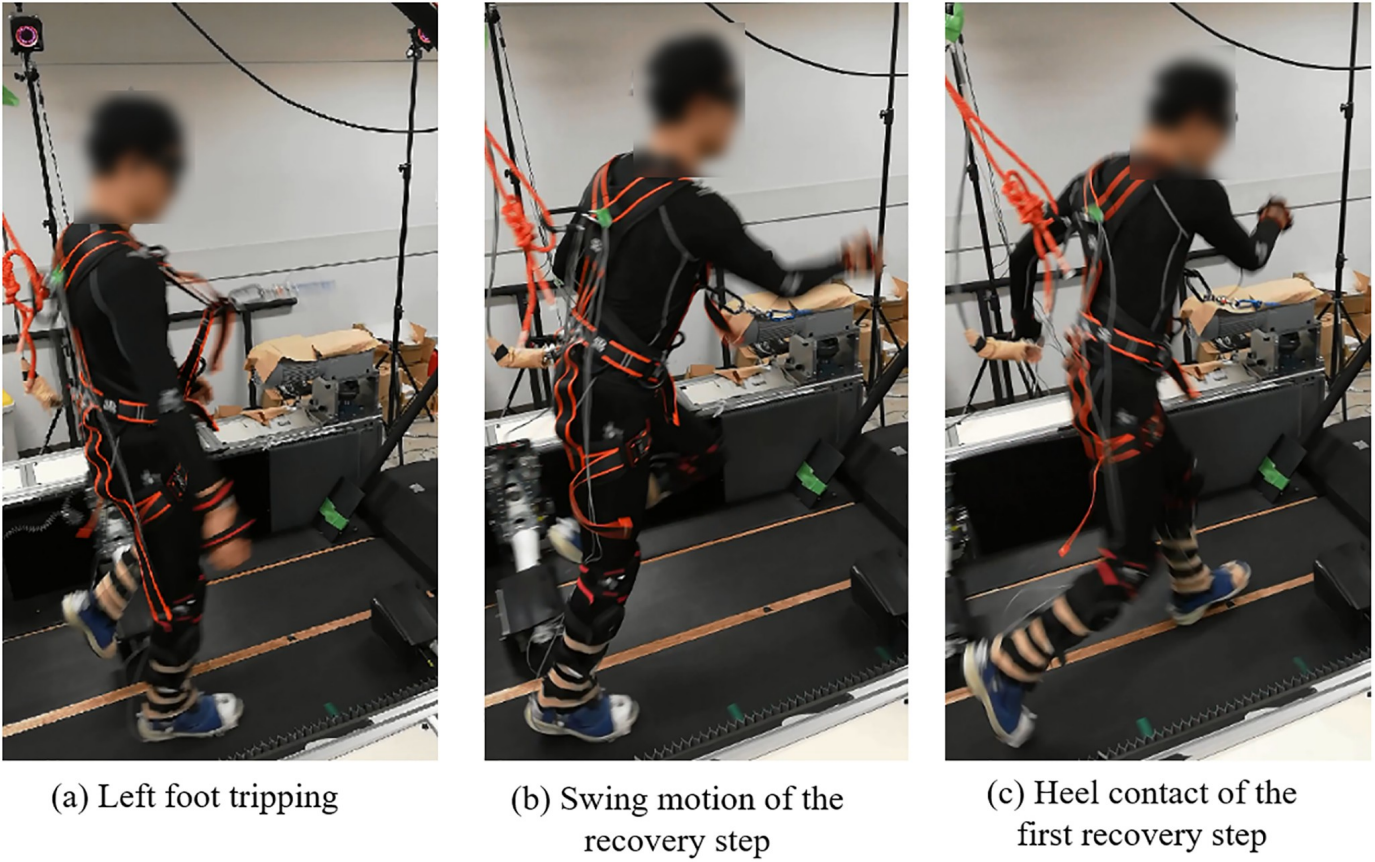
Recovery step after perturbation. (a) Left foot tripping (b) Swing motion of the recovery step (c) Heel contact of the first recovery step.

A total of 192 recovery trials were recorded and analyzed. Details of these trials can be found in S1 Table. The representative recovery motion is illustrated in Fig 2, where the subject is shown stepping over the tripping bar with the tripped leg.

The median and interquartile range (IQR) of the recovery parameters for each condition are summarized in Table 1. The mean values of some parameters seem different among the sets of conditions. The correlation coefficients of the recovery motion parameters are summarized in Table 2. Although the absolute value of the correlation coefficient is less than 0.4 for almost all parameters, the correlations of *Tr*_*ang*_ and *Hip*_*ang*_ between the FS and SS are large, at 0.69 and 0.78, respectively.

**Table 1:** Median and IQR of recovery motion parameters among conditions.

	Unit	Normal	Pull	Fast	Heavy
*Tr*_*ang*_ − *FS*	deg	37.63; 33.55–45.15	36.84; 33.78–43.48	42.30; 37.46–49.30	41.36; 36.44–47.84
*Hip*_*ang*_ − *FS*	deg	15.85; 11.52–19.85	15.65; 12.62–20.57	13.88; 10.67–17.28	15.06; 11.05–18.74
*CoMv* − *FS*	m/s	-0.39; -0.45–-0.34	-0.54; -0.61–-0.43	-0.44; -0.48–-0.32	-0.38; -0.46–-0.29
*St*_*length*_ − *FS*	m	0.62; 0.51–0.68	0.75; 0.66–0.80	0.67; 0.62–0.75	0.61; 0.54–0.69
*St*_*time*_ − *FS*	s	0.50; 0.47–0.54	0.47; 0.44–0.51	0.48; 0.46–0.50	0.50; 0.48–0.55
*Tr*_*ang*_ − *SS*	deg	28.39; 25.03–35.85	28.28; 24.57–33.98	31.87; 28.50–36.23	32.26; 26.07–34.75
*Hip*_*ang*_ − *SS*	deg	13.25; 8.71–18.65	12.38; 8.63–17.00	11.04; 8.69–17.04	11.27; 8.01–19.15
*CoMv* − *SS*	m/s	-0.21; -0.30–-0.15	-0.21; -0.26–-0.15	-0.24; -0.30–-0.20	-0.21; -0.26–-0.16
*St*_*length*_ − *SS*	m	0.64; 0.58–0.69	0.63; 0.58–0.71	0.71; 0.66–0.77	0.65; 0.62–0.69
*St*_*time*_ − *SS*	s	0.48; 0.45–0.52	0.48; 0.44–0.54	0.47; 0.43–0.49	0.49; 0.45–0.51
*Max*_*mom*_	Nm	0.04; 0.04–0.05	0.05; 0.04–0.06	0.04; 0.03–0.04	0.04; 0.04–0.05
*Mean*_*mom*_	Nm	0.02; 0.02–0.03	0.02; 0.02–0.03	0.02; 0.02–0.02	0.02; 0.02–0.03
*Ptime*_*mom*_	s	0.17; 0.15–0.38	0.13; 0.08–0.16	0.17; 0.12–0.37	0.16; 0.12–0.33
*HC*_*mom*_	Nm	0.01; 0.00–0.02	0.00; -0.01–0.01	0.01; 0.00–0.02	0.01; 0.00–0.02

**Table 2:** Correlation coefficients of recovery motion parameters.

	*Hip*_*ang*_ −*FS*	*CoMv* −*FS*	*St*_*length*_ −*FS*	*St*_*time*_ −*FS*	*Tr*_*ang*_ −*SS*	*Hip*_*ang*_ −*SS*	*CoMv* −*SS*	*St*_*length*_ −*SS*	*St*_*time*_ −*SS*	*Max*_*mom*_	*Mean*_*mom*_	*Ptime*_*mom*_	*HC*_*mom*_
*Tr*_*ang*_ − *FS*	0.33	-0.27	0.15	0.32	0.69	0.17	0.14	0.03	0.29	-0.01	0.10	0.35	0.14
*Hip*_*ang*_ − *FS*		-0.37	-0.07	0.25	0.34	0.78	-0.25	0.02	0.08	0.29	0.50	0.22	0.22
*CoMv* − *FS*			-0.34	-0.08	-0.21	-0.15	-0.13	0.11	-0.18	-0.34	-0.22	0.16	0.17
*St*_*length*_ − *FS*				-0.07	-0.02	-0.29	0.24	0.04	0.35	0.02	0.01	-0.11	0.00
*St*_*time*_ − *FS*					0.33	0.13	0.04	0.00	0.09	0.38	0.05	0.17	-0.18
*Tr*_*ang*_ − *SS*						0.17	0.09	0.23	0.26	0.09	0.04	0.21	-0.08
*Hip*_*ang*_ − *SS*							-0.40	-0.01	-0.19	0.09	0.31	0.26	0.26
*CoMv* − *SS*								-0.40	0.22	0.09	-0.23	-0.03	-0.11
*St*_*length*_ − *SS*									0.11	0.02	0.03	-0.05	-0.01
*St*_*time*_ − *SS*										0.04	0.10	-0.07	0.29
*Max*_*mom*_											0.35	-0.11	-0.28
*Mean*_*mom*_												0.16	0.26
*Ptime*_*mom*_													0.22

**Fig 3:**
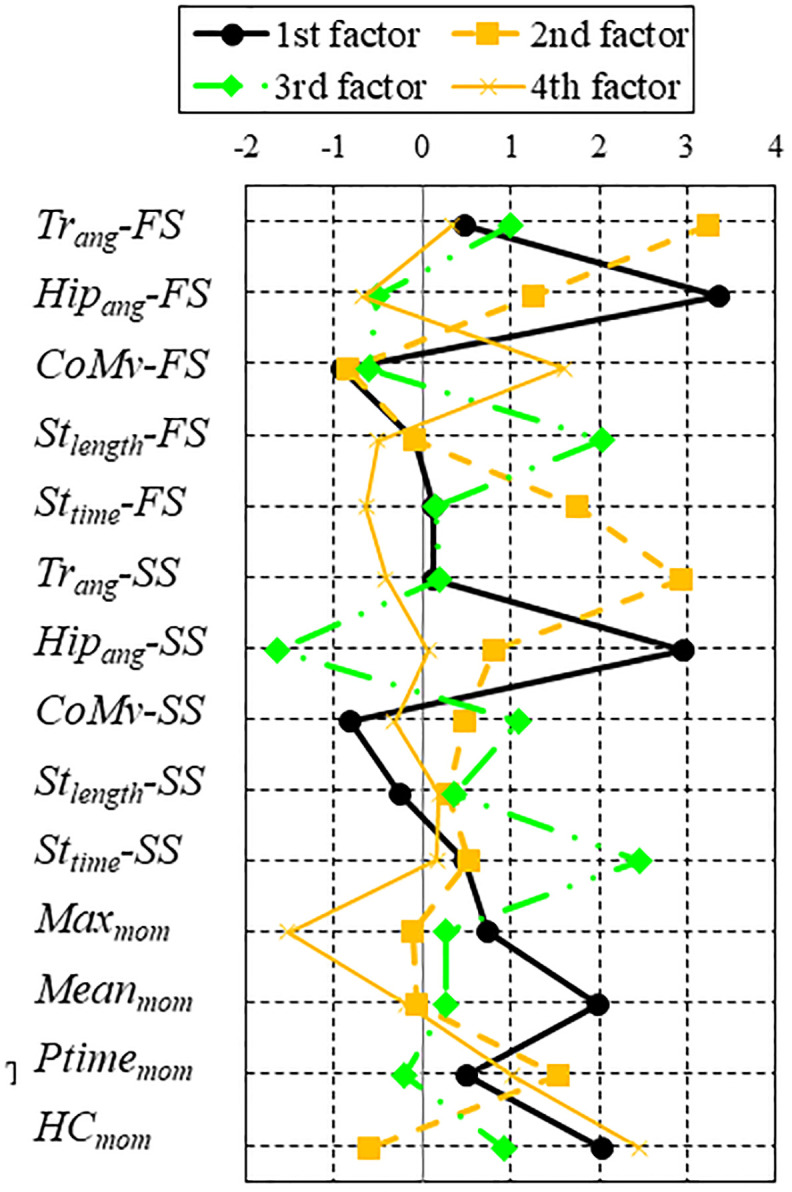
Factor loading.

**Fig 4:**
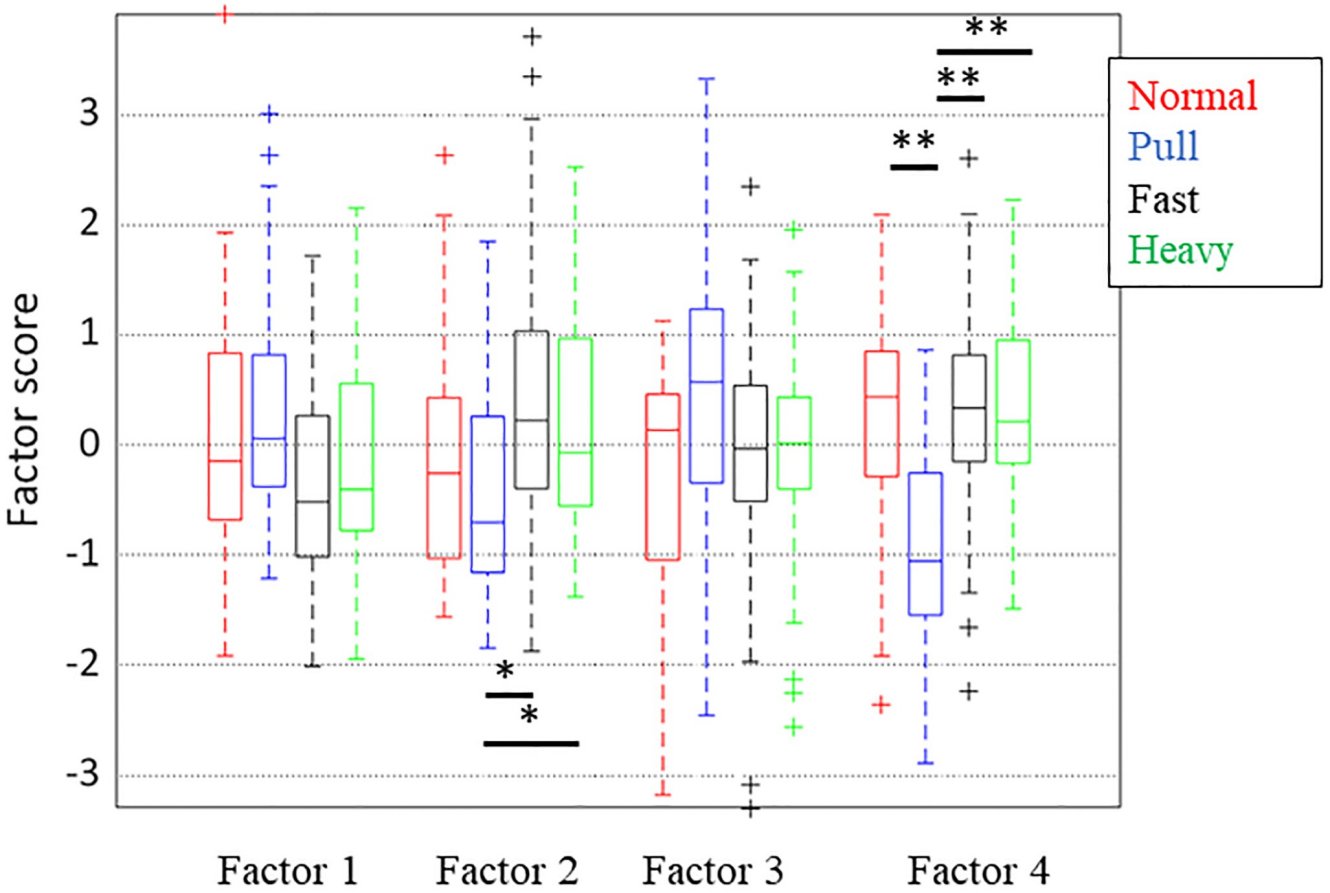
Factor score. (*: *p*< 0.05, **: *p*< 0.01).

**Fig 5:**
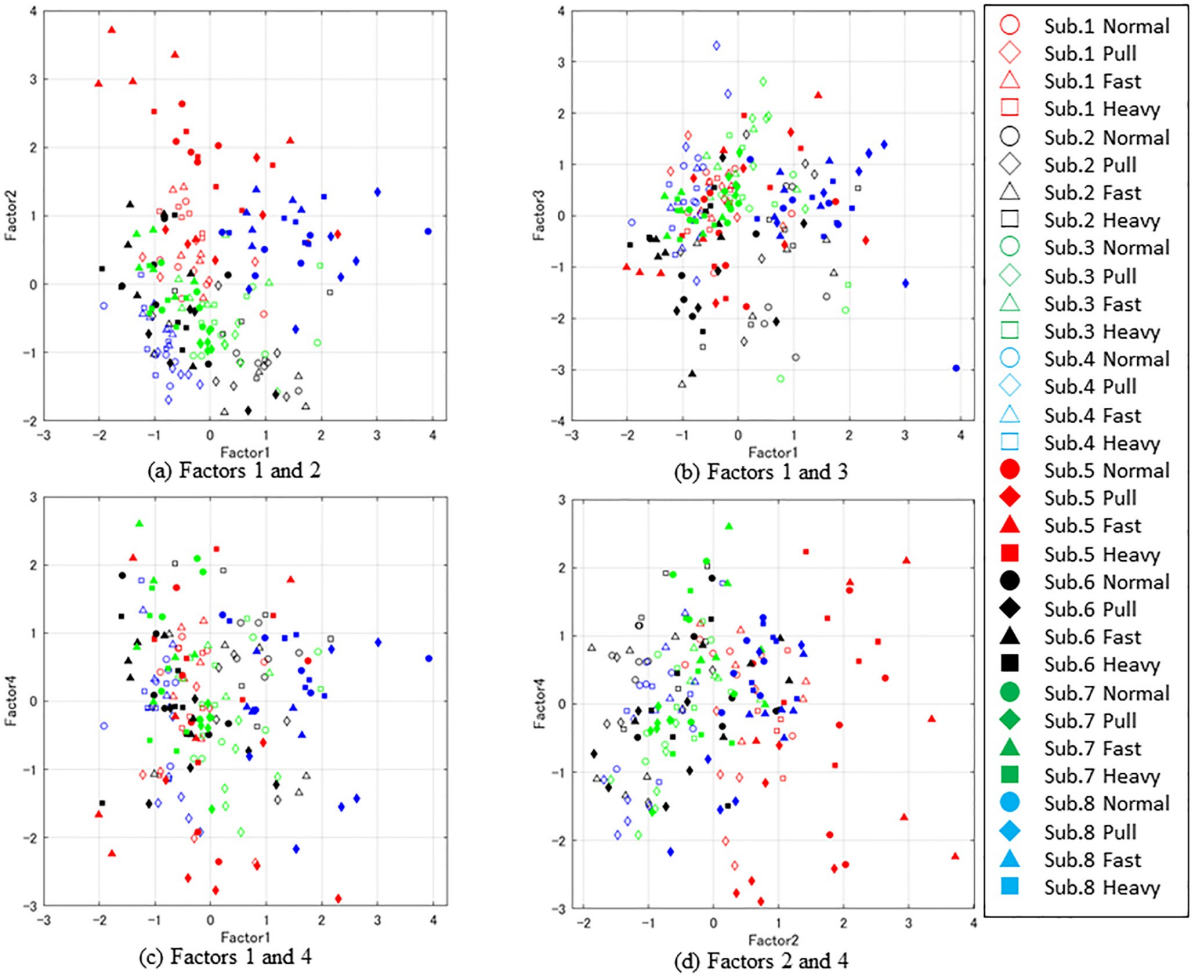
Distribution of factor scores. (a) Factors 1 and 2 (b) Factors 1 and 3 (c) Factors 1 and 4 (d) Factors 2 and 4.

Four factors with contribution ratios of 14.8%, 14.3%, 11.0%, and 9.8% were extracted as FA results in this study. The contribution ratio is 49.9%. The FA results are presented in Figs 3–5. The loading of each factor weighted by its contribution is described in Fig 3. The distribution of factor scores under each set of conditions is presented in Fig 4. Significant differences appear for the second and fourth factors. In the second factor, the pull condition significantly differed from those under the fast and heavy conditions. In the fourth factor, the pull condition significantly differed from those under all the other conditions. Fig 5 provides a scatter plot of the factor scores of all samples for each set of conditions and subject. The plots for each subject overlap, whereas the distributions of the subjects are sometimes condensed.

## Discussion

### Relationship between motion parameters and forward moment

A factor that has large factor loadings on both the motion and moment parameters indicates a relationship between them. In this experiment, the loadings of the first, second, and fourth factors became large at moment parameters such as *Max*_*mom*_, *Mean*_*mom*_, *Ptime*_*mom*_, and *HC*_*mom*_, as illustrated in Fig 3. The first, second, and fourth factors can be considered as the parameters that relate the motion and moment parameters because they also have large factor loadings to the motion parameters.

Based on Fig 3, motion parameter *Tr*_*ang*_ of both the FS and SS and moment parameters *Mean*_*mom*_ and *HC*_*mom*_ are related to the first factor. The trunk angle can be considered a parameter of the magnitude of the recovery motion [5]. A large first factor means that the trunk tilts forward, and the forward moment simultaneously increases. It is reasonable that the forward moment increases when the body inclines forward because of the large mass and inertia of the pelvis, torso, and trunk [32, 33]. Thus, the first factor could be considered a factor causing a larger forward fall motion.

Meanwhile, the second factor is represented as the *Hip*_*ang*_ for both the FS and SS. A larger hip angle indicates that the subject moved his thigh higher during the recovery step. Furthermore, the *Hip*_*ang*_ values are moderately related to *St*_*time*_ of the FS and *Ptime*_*mom*_. Thus, the second factor indicates the simultaneous occurrence of a larger hip flexion angle, longer step time, and longer duration before the timing of the peak moment. The hip flexion probably mitigates the forward moment because the thigh, whose mass is also large among the links of the human body [32], moves up in the forward position from the CoM. However, *Max*_*mom*_ and *Mean*_*mom*_, which are parameters of the magnitude of the forward moment, are not related to this factor. This means that the change of hip flexion pattern did not affect the peak value of forward moment.

The fourth factor is related to *CoMv* of the FS, *Max*_*mom*_, and *HC*_*mom*_. It is unusual that the signs of *Max*_*mom*_ and *HC*_*mom*_ are inverted, because they are both parameters that cause a larger forward moment. It appears that this factor represents some special aspects of the recovery motion, as mentioned in the subsection below. The third factor consists of *St*_*length*_ of the FS, *Tr*_*ang*_ of the SS, and *St*_*time*_. All these parameters are related to motion, and none of the moment parameters is included in this factor. Thus, this factor does not connect the motion and moment parameters.

### Difference among methods to add forward moment

In this study, the forward moment after tripping was artificially increased using several methods. The normalized moment parameters enabled comparison of the qualitative changes in recovery motion after tripping by compensating for the direct effects of additional mass and speed on the forward moment. Among these conditions, the pull condition significantly varied from the other conditions based on the fourth factor score, as shown in Fig 4. Similarly, the second factor of the pull condition significantly differed from those under the other conditions, except for the normal condition. However, no meaningful differences were found for the other pairs of conditions.

The first factor, which reflected the magnitude of the normalized forward moment of the first recovery phase, represents the increase in forward moment owing to an increase in body inclination. However, this factor does not differ significantly among the considered sets of conditions. It seems that the difference in the first factor score reflected a qualitative difference in the relationship between the forward moment and body inclination because the effects of the body mass and gait speed were compensated for by normalization. Thus, it was implied that the method of increasing the forward moment did not significantly affect the pattern of the recovery motion represented by the first factor.

The second factor, which relates the hip angle and time before the peak moment, differs slightly among the investigated sets of conditions. However, according to the pairwise comparison, the score under the normal condition did not differ significantly from those of under the fast and heavy conditions. This finding implies that the method used to increase the forward moment slightly affected the parameters related to the second factor. The magnitude of hip flexion during the recovery step may reflect the uniqueness of each subject, rather than the effect of the condition.

As mentioned above, the fourth factor consists of *CoMv* of FS, *Max*_*mom*_, and *HC*_*mom*_. A large negative fourth factor under the pull condition means that *Max*_*mom*_ becomes large, whereas *CoMv* and *HC*_*mom*_ are small, as shown in Fig 4. This combination of parameters implies that the forward moment temporarily increased after tripping owing to the pulling force. However, it was compensated for before the FS. Furthermore, this trend only occurred under the pull condition, which indicated the uniqueness of this condition compared to the other methods of increasing the forward moment.

### Limitations

In this study, an experiment was performed on a treadmill to control the conditions of tripping accurately. Furthermore, the pull condition is only possible on a treadmill. However, high acceleration and deceleration after tripping may decrease the physical similarity between overground and treadmill walking. Therefore, an investigation of the similarity of recovery motions between overground and treadmill walking is required.

In addition, only young healthy adults who have relatively high gait ability among humans participated in this experiment. They could compensate for the effect of the increased forward moment without qualitatively changing the recovery motion. For example, the elderly often adopt a different arm movement compared to young adults, focusing more on guarding their body rather than maintaining balance, as noted by Roos et al. (2008) [34]. Thus, it is possible that the effects of these conditions would increase in the elderly or patients with gait impairment. Understanding the recovery strategy against tripping remains a subject for future study.

Based on the results, the contribution ratio of the FA was not very high, which means that only half of the sample deviations could be explained by our model. Thus, other parameters that affect human behavior during recovery motion should be considered.

## Conclusion

To investigate the effects of the forward moment on the pattern of the recovery motion after tripping, the relationships between the motion and moment parameters were analyzed. The forward moment was artificially increased using several methods, i.e., pulling a torso in the forward direction, increasing gait speed, and increasing body mass, and a tripping experiment was performed on a treadmill. The qualitative differences in the recovery motion among the methods of increasing the forward moment were extracted by analyzing the normalized motion and moment parameters.

The results of the FA indicated a factor that links the maximum trunk inclination and relative magnitude of the normalized forward moment. The distribution of the factor scores revealed that increasing the forward moment using the different methods did not qualitatively change the recovery motion. Thus, the recovery motion after tripping was robust against the increase in forward moment in this study, regardless of the method used to increase the forward moment. In particular, the effects of the differences in motion among the considered sets of conditions were less than those of the individual conditions.

However, another factor indicated the uniqueness of the pull condition from the perspective of motion characteristics. The increase in the forward moment applied by the pulling force temporarily increased the forward moment, but it could be compensated for before the first recovery step. This trend was extracted as an independent factor, and the scores of the pull condition differed significantly from those of the other conditions. Thus, the uniqueness of the pull condition probably enables the investigation of the recovery motion structure; however, it may not be suitable for reproducing the natural reaction motion.

This study elucidated the mechanism of recovery motion and expandability of the recovery motion pattern against a variable forward moment. The results of this study will facilitate faller recovery estimation and mitigation motion. The observations of the recovery motion of various subjects with different attributes will improve the knowledge of the fall mechanism.

## Supporting information

S1 Table Parameters for factor analysis. Calculated parameters of each trial of each subject. This table was used to perform factor analysis.
(CSV)
